# Rifaximin disc diffusion test for *in vitro* susceptibility testing of *Clostridium difficile*

**DOI:** 10.1099/jmm.0.028571-0

**Published:** 2011-08

**Authors:** Steliana Huhulescu, Ulrich Sagel, Anita Fiedler, Verena Pecavar, Marion Blaschitz, Guenther Wewalka, Franz Allerberger, Alexander Indra

**Affiliations:** 1 Austrian Agency for Health and Food Safety (AGES), Institute for Medical Microbiology, Hygiene, Waehringerstrasse 25a, A-1096 Vienna, Austria; 2 Paracelsus Medical University, Institute for Medical Microbiology, Hygiene and Infectious Diseases, Muellner Hauptstrasse 48, A-5020 Salzburg, Austria

## Abstract

Rifaximin is a rifampicin derivative, poorly absorbed by the gastro-intestinal tract. We studied the *in vitro* susceptibility to rifamixin of 1082 *Clostridium difficile* isolates; among these,184 isolates from a strain collection were tested by an in-house rifaximin disc (40 µg) diffusion test, by an in-house rifaximin broth microdilution test, by rifampicin Etest and by *rpoB* gene sequencing. In the absence of respective CLSI or EUCAST MIC breakpoints for rifaximin and rifampicin against *C. difficile* we chose MIC ≥32 µg ml^−1^ as criterion for reduced *in vitro* susceptibility. To further validate the disc diffusion test 898 consecutive clinical isolates were analysed using the disc diffusion test, the Etest and *rpoB* gene sequence analysis for all resistant strains. Rifaximin broth microdilution tests of the 184 reference strains yielded rifaximin MICs ranging from 0.001 (*n* = 1) to ≥1024 µg ml^−1^ (*n* = 61); 62 isolates showed a reduced susceptibility (MIC ≥32 µg ml^−1^). All of these 62 strains showed *rpoB* gene mutations producing amino acid substitutions; the rifampicin- and rifaximin-susceptible strains showed either a wild-type sequence or silent amino acid substitutions (19 strains). For 11 arbitrarily chosen isolates with rifaximin MICs of >1024 µg ml^−1^, rifaximin end-point MICs were determined by broth dilution: 4096 µg ml^−1^ (*n* = 2), 8192 µg ml^−1^ (*n* = 6), 16 384 µg ml^−1^ (*n* = 2) and 32 678 µg ml^−1^ (*n* = 1). Rifampicin Etests on the 184 *C. difficile* reference strains yielded MICs ranging from ≤0.002 (*n* = 117) to ≥32 µg ml^−1^ (*n* = 59). Using a 38 mm inhibition zone as breakpoint for reduced susceptibility the use of rifaximin disc diffusion yielded 59 results correlating with those obtained by use of rifaximin broth microdilution in 98.4 % of the 184 strains tested. Rifampicin Etests performed on the 898 clinical isolates revealed that 67 isolates had MICs of ≥32 µg ml^−1^. There were no discordant results observed among these isolates with reduced susceptibility using an MIC of ≥32 µg ml^−1^ as breakpoint for reduced rifampicin susceptibility and a <38 mm inhibition zone as breakpoint for reduced rifaximin susceptibility. The prevalence of reduced susceptibility was 7.5 % for all isolates tested. However, for PCR ribotype 027 the prevalence of reduced susceptibility was 26 %. Susceptibility testing in the microbiology laboratory therefore could have an impact on the care and outcome of patients with infection. Our results show that rifaximin – despite its water-insolubility – may be a suitable candidate for disc diffusion testing.

## Introduction

*Clostridium difficile* is a spore-forming Gram-positive anaerobic bacterium and a major cause of nosocomial and community-acquired diarrhoea (Wiström *et al.*, 2001). Metronidazole and oral vancomycin are the main antibiotics used to treat *C. difficile* infection (CDI). In 2005, in Austria, oral rifaximin was licensed for ‘*treatment of gastrointestinal diseases caused or partially caused by rifaximin-susceptible bacteria, e.g. gastrointestinal infections, pseudomembranous colitis due to C. difficile, hepatic encephalopathy, small bowel bacterial overgrowth and diverticulitis*’ (Willerroider, 2009). However, to our knowledge, no guidelines have yet been published to test *in vitro* susceptibility of *C. difficile* to rifaximin. Two study groups previously used ≥32 mg l^−1^ (O’Connor *et al.*, 2008; Jiang *et al.*, 2010) as breakpoint for resistance using agar dilution testing, a method not widely employed in routine testing of clinical isolates. Jiang *et al.* (2000) reported high rifaximin concentrations (4000 to 8000 µg g^−1^) in stools 3 days after a single oral administration. Johnson *et al.* (2007) reported on the possible prevention of recurrence of CDI by administering rifaximin immediately after completion of the last course of vancomycin therapy of CDI.

In Austria, rifaximin is widely used to treat CDI, and microbiological laboratories often receive requests for *in vitro* susceptibility testing of clinical *C. difficile* isolates against this substance. The aim of this study was to evaluate whether the disc diffusion method can be applied to test *in vitro* susceptibility of *C. difficile* to rifaximin.

## Methods

### 

#### Micro-organisms.

One hundred and eighty-four *C. difficile* isolates were obtained from the reference strain collection of the Austrian national *C. difficile* reference centre, including ATCC strain 9689, and 50 strains previously provided by Leeds University Hospital (UK), 3 strains from the Public Health Laboratory Maribor (Slovenia) and 24 strains from Leiden University Hospital (Netherlands). In addition, 898 non-duplicate clinical isolates were tested, cultured in 21 Austrian medical laboratories in 2009.

All 1082 isolates were stored at −80 °C in cryobank tubes (Mast Diagnostics) until testing. Isolates were recultivated on Columbia blood agar plates (bioMérieux); all were tested for PCR ribotype (RT) (Indra *et al.*, 2008), toxin A (A) (van den Berg *et al.*, 2004), toxin B (B) (Kato *et al.*, 1991, 1999), binary toxin (BT) (Stubbs *et al.*, 2000) and *tcdC* deletion (Spigaglia & Mastrantonio, 2002). Tables 1 and 2 list the PCR ribotypes of these isolates. All 1082 isolates except one (PCR ribotype 010) produced at least toxin B; 149 of them (14 %) were A/B/BT positive and belonged to the following ribotypes: 027 (*n* = 99), 078 (*n* = 10), 176 (*n* = 9), 023 (*n* = 5), 080 (*n* = 3), 126 (*n* = 2), 419 (*n* = 2), 045 (*n* = 2), 411 (*n* = 2), 429 (*n* = 2), 250 (*n* = 1), 413 (*n* = 1), 018 (*n* = 1), 515 (*n* = 1), 344 (*n* = 1), 605 (*n* = 1), 606 (*n* = 1), 616 (*n* = 1), 654 (*n* = 1), 655 (*n* = 1), 656 (*n* = 1), 657 (*n* = 1), 658 (*n* = 1). If possible nomenclature was used according to that given by the John Brazier’s laboratory, due to the higher quality of resolution gained with capillary sequencer-based PCR-ribotyping (Indra *et al.*, 2008) strains not assigned by John Brazier’s laboratory were given numbers starting with 400.

**Table 1.** Distribution of ribotypes among 184 *C. difficile* isolates from the strain collection of the Austrian national *C. difficile* reference centre

Ribotype	No. of strains	Percentage
027	26	14.13
053	16	8.70
001	7	3.80
014/0	6	3.26
005	3	1.63
056	3	1.63
078	3	1.63
239	3	1.63
408	3	1.63
002/2	2	1.09
012	2	1.09
017	2	1.09
020	2	1.09
029	2	1.09
031	2	1.09
043	2	1.09
046	2	1.09
404	2	1.09
503	2	1.09
510	2	1.09
Other*	92	50

* Represented by one isolate each: 002/0, 002/1, 003, 004, 006, 007, 009, 010, 014, 015, 016, 018, 019, 023, 025, 026, 033, 035, 036, 037, 039, 040, 042, 045, 047, 049, 050, 051, 052, 054, 055, 057, 058, 060, 062, 063, 064, 066, 067, 068, 070, 072, 075, 076, 077, 079, 080, 081, 083, 084, 085, 087, 094, 095, 106, 115, 117, 118, 122, 126, 131, 153, 169, 174, 201, 209, 212, 220, 411, 413, 434, 441, 444, 448, 466, 497, 504, 523, 524, 539, 542, 548, 622, 627, 633, 643, 649, 650, 651, 652, 653, 654.

**Table 2.** Distribution of ribotypes among 898 clinical *C. difficile* isolates from 2009

Ribotype	No. of strains	Percentage
053	198	22
014/0	81	9.0
027	73	8.1
002/2	35	4.0
001	28	3.1
005	26	2.8
‘Infrequent’ ribotypes (*n* = 10–25)*	161	17.8
‘Rare’ ribotypes (*n* = <10)†	296	33.2

* 010, 012, 018, 020, 029, 078, 241, 408, 600.

† 003, 009, 014/5, 015, 017, 019, 023, 025, 026, 031, 043, 045, 046, 049, 054, 056, 066, 070, 080, 081, 087, 126, 153, 176, 203, 205, 206, 207, 208, 209, 211, 212, 220, 232, 236, 237, 239, 250, 403, 404, 405, 411, 413, 415, 419, 425, 429, 430, 431, 432, 434, 438, 439, 440, 441, 442, 448, 449, 451, 453, 457, 470, 472, 477, 481, 483, 484, 486, 492, 495, 496, 498, 499, 500, 501, 502, 503, 504, 505, 507, 508, 510, 512, 514, 515, 516, 518, 519, 520, 523, 525, 526, 530, 531, 532, 535, 537, 542, 548, 549, 601, 602, 603, 604, 605, 606, 607, 608, 609, 610, 611, 612, 613, 614, 615, 616, 617, 618, 619, 620, 621, 622, 623, 624, 625, 626, 627, 628, 629, 630, 631, 632, 633, 634, 635, 636, 637, 638, 639, 640, 641, 642, 643, 644, 645, 646, 647, 648, 655, 656, 657, 658.

#### Antimicrobial agents and susceptibility testing.

The 184 isolates from the strain collection were tested by an in-house rifaximin disc (40 µg) diffusion test (Oxoid; custom-made product), by an in-house rifaximin broth microdilution test and by rifampicin Epsilon-test (Etest) (bioMérieux). The 898 clinical isolates from 2009 were tested only by disc diffusion test and rifampicin Etest (bioMérieux). Rifaximin discs were custom-made (Oxoid) by Gebro Pharma. The disc diffusion tests were performed on Brucella blood agar plates supplemented with 5 mg haemin l^−1^ and 1 mg vitamin K l^−1^ (Oxoid).

The rifaximin broth microdilution test was performed as follows. Rifaximin was purchased as a powder from Alfa Wassermann. Sterile stock solutions were prepared according to the instructions of CLSI for testing of anaerobes (Clinical and Laboratory Standards Institute, 2007). In short, rifaximin was dissolved in methanol and then diluted in 0.9 % saline solution. Serial twofold dilutions in ATB S-medium containing menadione (vitamin K_3_) at 0.5 mg l^−1^ and haemin at 15 mg l^−1^ (bioMérieux) were prepared for rifaximin concentrations covering a range from 0.000125 to 1024 µg ml^−1^; 96-well microdilution plates were filled with ATB S-medium (bioMérieux) containing the respective antibiotic concentrations. The antibiotic stocks were freshly prepared on the day of testing. For inoculum preparation, test organisms were cultured for 48 h anaerobically on Columbia blood agar plates (bioMérieux) at 37 °C; bacteria were suspended in 0.9 % saline solution to yield McFarland 0.5 and diluted 1 : 10 into the medium, so that the final test concentration of bacteria was approximately 1×10^6^ c.f.u. ml^−1^. Minimal inhibitory concentration (MIC) was defined as the lowest concentration at which no growth was observed after incubation for 48 h at 37 °C in an anaerobic atmosphere using anaerobic jars and GasPak (BD). Growth controls were performed by inoculation of antibiotic-free medium with an aliquot of the primary inoculum at a concentration of 10^5^ c.f.u. per well; purity testing was performed by transferring an aliquot of 10 µl onto two Columbia blood agar plates with incubation under aerobic and anaerobic conditions, respectively.

Rifampicin Etests (MIC range 0.002–32 µg ml^−1^) were performed according to the manufacturer’s instructions (bioMérieux) using Brucella blood agar plates supplemented with haemin (5 mg l^−1^) and vitamin K (1 mg l^−1^) (Oxoid).

In the absence of respective CLSI or EUCAST MIC breakpoints for rifaximin and rifampicin against *C. difficile* we chose MIC ≥32 µg ml^−1^ as criterion for reduced *in vitro* susceptibility, as suggested by O’Connor *et al.* (2008) and Jiang *et al.* (2010). Setting of zone diameter breakpoints for rifaximin susceptibility testing by disc (40 µg) diffusion was performed according to the recommendations of Turnidge & Paterson (2007).

Statistical analyses were performed with stata/ic 10.1 (StataCorp).

#### Detection of single-nucleotide polymorphisms (SNPs) within the *rpoB* gene.

DNA was extracted from cultures using the MagNA Pure Compact (Roche Diagnostics) according to the producer’s manual to a final volume of 50 µl. Primers RifFOR (5′-CAAGATATGGAAGCTATAAC-3′) and RifREVlang (5′-GTGATTCTATAAATCCAAATTC-3′) were used in PCRs containing 25 µl HotStar *Taq* Master Mix (Qiagen), 5 µl (5 pmol µl^−1^) of each primer, 13 µl water and 2 µl DNA. Amplification was performed in a PCR thermocycler (15 min 96 °C, 30 cycles of 1 min 94 °C, 1 min 52 °C and 1 min 72 °C, and finally 10 min 72 °C). PCR products were cleaned up with *Escherichia coli* exonuclease I, and shrimp alkaline phosphatase (Fermentas) according to the manufacturer’s instructions.

Sequencing PCR containing 2 µl Big-Dye-Mix (Applied Biosystems), 1 µl Sequencing Buffer (Applied Biosystems) 4 µl water, 1 µl RifFOR or RifREVlang primer (10 pmol^−1^) and 2 µl DNA was performed in a commercial PCR thermocycler (1 min 96 °C, 30 cycles of 20 s 96 °C, 20 s 50 °C and 4 min 60 °C). The amplified products were cleaned up with Centri Sep 96-well plates or Centri Sep 8 well strips (Applied Biosystems) for dye terminator clean-up according to the manufacturer’s manual. Samples were analysed in an ABI 3130 genetic analyser (Applied Biosystems) with 36 cm capillary loaded with a POP7 gel (Applied Biosystems).

Sequences were analysed for the presence of SNPs within the *rpoB* gene using Kodon (Applied Maths) version 3.5 by aligning the samples to the *rpoB* gene sequence of a reference wild-type *C. difficile* strain (CD630; NC_009089) downloaded from the NCBI database.

## Results

### Results for reference strains

**Fig. 1.**
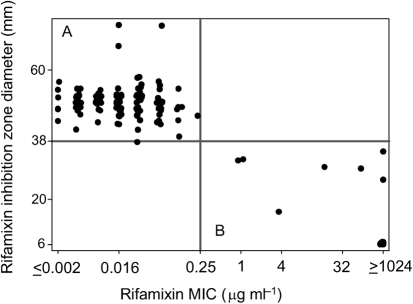
Rifaximin disc (40 µg) diffusion test results of 184 *C. difficile* reference strains compared to MICs obtained by the rifaximin broth microdilution test. All measured ‘sensitive’ *C. difficile* strains (*n* = 122) are grouped in quadrant A, in contrast to the 62 strains identified as ‘reduced susceptibility’ in quadrant B.

Rifaximin disc (40 µg) diffusion testing of the 184 reference strains yielded inhibition zone diameters ranging from 6 mm to 74 mm. Rifaximin broth microdilution tests of the 184 reference strains yielded rifaximin MICs ranging from 0.001 (*n* = 1) to ≥1024 µg ml^−1^ (*n* = 61); 62 strains showed reduced susceptibility with an MIC of at least 32 µg ml^−1^. The MIC_50_ of rifaximin was 0.032 µg ml^−1^ and the MIC_90_ was ≥1024 µg ml^−1^; the inhibition diameters in comparison to the results obtained by broth microdilution test are summarized in Fig. 1. The most common *rpoB* mutation found within this group was R505K (*n* = 46); other mutations were H502N+R505K (*n* = 6), H502Y (*n* = 2), H502N (*n* = 2) and one each of H502L, H502N+A555A, L487F+H502Y, R505K+I548M, D492V and S550F.

For 11 arbitrarily chosen strains with rifaximin MICs of >1024 µg ml^−1^, rifaximin end-point MICs were determined by broth dilution: 4096 µg ml^−1^ (*n* = 2: strains 2228 and 2639), 8192 µg ml^−1^ (*n* = 6: strains 2236, 2285, 2312, 2383, 2816 and 3025), 16 384 µg ml^−1^ (*n* = 2: strains 3091 and 910016) and 32 678 µg ml^−1^ (*n* = 1: strain 3098). These 11 strains yielded the following mutations in their *rpoB* genes: R505K (strains 2228, 2236, 2285, 2312, 2383, 2639, 3091 and 910016), L487F+H502Y (strain 2816), H502N+R505K (strain 3025), R505K+I548M (strain 3098).

Rifampicin Etests on these 184 *C. difficile* strains yielded MICs ranging from ≤0.002 (*n* = 117) to ≥32 µg ml^−1^ (*n* = 59); 59 strains had reduced susceptibility (MIC ≥32 µg ml^−1^). The MIC_50_ of rifampicin was ≤0.002 µg ml^−1^ and the MIC_90_ was ≥32 µg ml^−1^; all 59 strains showed mutations in the *rpoB* region (R505K (*n* = 46), H502N+R505K (*n* = 6), H502Y (*n* = 2) and one each of H502L, S550F, H502N+A555A, L487F+H502Y, R505K+I548M). In 181 (98.4 %) of the 184 strains tested, the use of the rifampicin Etest yielded results (reduced susceptibility or non-resistant) in accordance with those obtained by the rifaximin broth microdilution test. Discordant results (rifaximin vs rifampicin) were found for three strains. Strain 2347/655 (showing an H502N *rpoB* mutation) and 3153/PCR ribotype 002/0 (showing a D492V *rpoB* mutation) had a rifaximin broth microdilution MIC of ≥1024 µg ml^−1^ and a rifampicin Etest MIC of 0.064 µg ml^−1^ and 1 µg ml^−1^, respectively. Strain Lee047/PCR ribotype 047 had a rifaximin broth microdilution MIC of 64 µg ml^−1^ and a rifampicin Etest MIC of 0.25 µg ml^−1^. Molecular analyses of these strains revealed a mutation in the *rpoB* gene giving an H502N amino acid substitution (Table 3).

**Table 3.** Amino acid substitutions detected by *rpoB* sequence analysis in *C. difficile* strains with discordant results

Isolate ID no.	*rpoB* mutation*	Rifampicin MIC (µg ml^−1^)	Rifaximin MIC (µg ml^−1^)	Rifaximin DD† inhibition zone (mm)
2203	H502N	0.5	1	32
2347	H502N	0.064	≥1024	34
2663	D492N	0.064	1	36
3018	S550Y	0.016	1	32
3109	T501T, L506L, G510G, G512G, F521F, E541E, K556K	0.125	16	30
3141	S475S, F481F, D492D, T501T, A508A, G510G, T539T, K556K	4	4	16
3153	D492V	1	≥1024	26
Lee047	H502N	0.25	64	30

* Resulting amino acid substitution shown.

† DD, disc diffusion.

Using an inhibition zone of <38 mm as breakpoint for reduced susceptibility, the use of rifaximin disc diffusion yielded results correlating with those received by the use of rifaximin broth microdilution in 180 (97.8 %) of the 184 strains tested. Strain 2203/PCR ribotype 053 and 3018/PCR ribotype 018 had rifaximin inhibition zones of 32 mm and rifaximin broth microdilution MICs of 1 µg ml^−1^ and showed an H502N or S550Y *rpoB* gene mutation, respectively. Strain 3109/PCR ribotype 002/0 had a rifaximin inhibition zone of 30 mm and a rifaximin broth microdilution MIC of 16 µg ml^−1^ and showed a number of silent mutations (T501T, L506L, G510G, G512G, F521F, E541E, K556K) in the *rpoB* gene. Strain 3141/PCR ribotype 539 had a rifaximin inhibition zone of 16 mm and a rifaximin broth microdilution MIC of 4 µg ml^−1^, and also showed a number of silent mutations (S475S, F481F, D492D, T501T, A508A, G510G, T539T, K556K) (Table 3).

The 58 strains with reduced susceptibility to rifampicin and rifaximin showed *rpoB* gene amino acid substitutions as follows: R505K (*n* = 46), H502N+R505K (*n* = 5), H502Y (*n* = 2) and one each of H502L, S550F, H502N+A555A, L487F+H502Y and R505K+I548M.

Of the 117 rifampicin- and rifaximin-susceptible strains with MICs of ≤0.002 µg ml^−1^, 98 (84 %) showed no point mutations in *rpoB*; 19 showed only silent amino acid substitutions as follows: T501T+L506L+G510G+G512G+F521F+E541E+K556K (*n* = 11), A555A (*n* = 6), L500L (*n* = 2).

Correlation of rifampicin MICs obtained by broth dilution with rifaximin disc diameters was tested by Kendall’s tau-b correlation coefficient: −0.42 (*P*<0.001 for the hypothesis that both parameters were independent).

### Results for clinical isolates

**Fig. 2.**
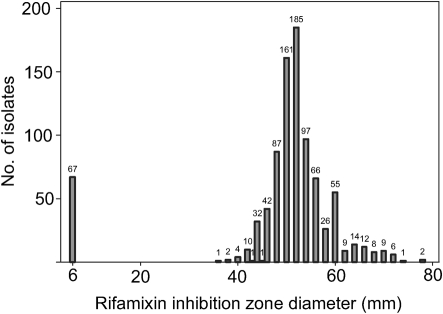
Rifaximin disc (40 µg) diffusion testing performed on 898 clinical *C. difficile* isolates yielding inhibition zone diameters ranging from 6 mm (zero inhibition) to 78 mm.

Rifaximin disc (40 µg) diffusion testing performed on 898 clinical *C. difficile* isolates yielded inhibition zone diameters ranging from 6 mm (*n* = 67) to 78 mm (*n* = 2) (Fig. 2). Using an inhibition zone <38 mm as breakpoint for reduced susceptibility a total of 68 strains with reduced susceptibility were identified.

Rifampicin Etests performed on the 898 isolates revealed that 67 isolates had MICs of ≥32 µg ml^−1^ for rifampicin and that 819 had MICs of ≤0.002 µg ml^−1^; 12 isolates (1.3 %) exhibited rifampicin MICs between these extremes. By rifaximin disc diffusion test, the 67 strains with reduced rifampicin susceptibility exhibited no inhibition zone. No discordant results were observed among these 67 isolates with reduced susceptibility using an MIC of ≥32 µg ml^−1^ as breakpoint for reduced rifampicin susceptibility and a <38 mm inhibition zone as breakpoint for reduced rifaximin susceptibility. Isolate 2663/PCR ribotype 053 had a rifampicin Etest MIC of 0.064 µg ml^−1^ and a rifaximin inhibition zone of 36 mm; molecular analyses revealed a D492N mutation in the *rpoB* gene (Table 3).

## Discussion

All the rifamycins are semisynthetic derivatives of rifamycin B, a fermentation product of *Amycolatopsis mediterranei*, formerly named *Streptomyces mediterranei*. Rifamycin B exerts poor antimicrobial activity, but is easily produced and readily converted chemically into rifamycin S, from which most active derivatives are prepared (Parenti & Lancini, 2003). Rifaximin is a semi-synthetic derivative of rifamycin S formulated for oral administration. In the 1980s it was only available in Italy, but today it is marketed worldwide mainly for the treatment of gastrointestinal infections and the treatment of chronic hepatic encephalopathy (Corazza *et al.*, 1988; Festi *et al.*, 1992). Jiang *et al.* (2000) reported high rifaximin concentrations (4000 to 8000 µg g^−1^) in stools 3 days after single oral administration.

Although adequate antimicrobial drug concentrations should be achieved by the high dosage of the non-absorbable rifaximin, the constraints imposed by possible drug resistance (O’Connor *et al.*, 2008) often impel clinicians to request *in vitro* susceptibility testing of *C. difficile* isolates from patients not responding to therapy. Our results on 11 arbitrarily chosen isolates from the group with reduced susceptibility to rifaximin (MIC >1024 µg ml^−1^) showed MICs ranging from 4096 to 32 678 µg ml^−1^, so that a breakpoint for resistance between >32 µg ml^−1^ and 1024 µg ml^−1^ can be considered for the future.

We evaluated a rifaximin disc diffusion test and found it substantially equivalent to an in-house rifaximin broth microdilution test. The level of performance considered acceptable for US Food and Drug Administration (FDA) clearance in premarket notification of commercial antimicrobial susceptibility systems is (among others) >89.9 % categorical agreement (same susceptible, intermediate or resistant classification), ≤1.5 % very major errors (false susceptibility based on the number of resistant organisms) and ≤3 % major errors (false resistance based on the number of susceptible isolates) (Richter & Ferraro, 2007). Accepting an MIC ≥32 µg ml^−1^ as criterion for reduced rifaximin susceptibility, a rifaximin inhibition zone of <38 mm would represent a valid resistance breakpoint in the 40 µg disc diffusion test described here.

We also found good correlation between the rifaximin and rifampicin susceptibility testing results. The number of discordant results (*n* = 8) concerning supposed resistance based on the rifampicin Etest and rifaximin disc diffusion test on *C. difficile* isolates could be seen as an argument to introduce the criterion ‘intermediate’ for rifampicin susceptibility testing. If we take the EUCAST breakpoints for staphylococci (S≤0.06/R>0.5 µg ml^−1^) for the rifampicin Etest, the correlation would increase to 99.45 % (five discordant results) (EUCAST, 2010). Our suggestions for preliminary breakpoints between susceptible, indeterminate and resistant are summarized in Table 4 for the Etest and the broth microdilution method to be validated in future studies. In our opinion no reliable intermediate breakpoint result can be given for the disc test; however, all isolates in question showed *rpoB* mutations yielding an amino acid change.

**Table 4.** Suggested preliminary breakpoints

	Rifaximin DD* inhibition zone (mm)	Rifaximin MIC (µg ml^−1^)	Rifampicin MIC (µg ml^−1^)
Susceptible	≥38	≤0.25	≤0.06
Intermediate	–	0.5–16	0.012–16
Resistant	<38	≥32	≥32

* DD, disc diffusion.

Rifamycin resistance is commonly the result of a mutation that alters the β-subunit of RNA polymerase, reducing its binding affinity for rifamycins (Struelens, 2003). This study found *rpoB* mutations in all of the 62 strains showing reduced susceptibility with the rifaximin broth microdilution test. Interestingly, only H502N and D492V mutations showed discordant results between the rifampicin Etest and the rifaximin broth microdilution test in the 184 strains analysed, indicating a potential connection between mutation in the *rpoB* region and the efficacy of the antibiotic used. Future studies would enable examination of this potential connection.

Our molecular investigations confirmed mutations resulting in amino acid substitutions in RpoB for all eight isolates with discordant results with reduced susceptibility to rifaximin and rifampicin. Interestingly, two strains showing seven or eight silent mutations also showed reduced susceptibility with the disc diffusion test, but not with any of the other methods tested. Since no other mutation could be found in the *rpoB* gene a mutation in another gene influencing the activity of rifaximin has to be taken into consideration. Studying *in vitro* activity of rifaximin against *C. difficile*, Ripa *et al.* (1987) previously postulated the occurrence of chromosomal resistance against rifaximin caused by mutation. In agreement with these authors, we found a good correlation between rifaximin and rifampicin susceptibility testing *in vitro*, underpinning the data obtained by O’Connor *et al.* (2008). However, these results seem to be in contrast to the findings of Jiang *et al.* (2010), who could not predict rifaximin resistance by doing only rifampicin resistance testing. Comparison of our data and those of O’Connor *et al.* (2008) with the study by Jiang *et al.* (2010) suggests that the use of acetone as solvent for rifaximin and rifampicin by Jiang’s group is the reason for the differences. To answer this question future studies should be undertaken. However, in our opinion, *in vitro* susceptibility testing of rifampicin can be used to predict resistance to rifaximin when done according to our method.

Rifaximin exhibits high activity against *C. difficile* *in vitro*, with a very low MIC_50_ and a very high MIC_90_ (0.032 µg ml^−1^ and 1024 µg ml^−1^, respectively). By using our suggested breakpoints (Table 4) as criteria for *in vitro* resistance, the prevalence of resistance was 7.5 % for all clinical isolates tested. Among the so-called hypervirulent ribotype RT 027, the prevalence of resistance was as high as 26 %. These data suggest that the development of resistance is in some way dependent on the PCR ribotype investigated; this is in concordance with general findings that some strains can become resistant more easily than others, e.g. *C. difficile* PCR ribotype 027, *Mycobacterium tuberculosis* spoligotype Beijing.

Susceptibility testing in the microbiology laboratory therefore could have an impact on the care and outcome of patients with infection (Doern *et al.*, 1994). The rifaximin agar dilution and broth microdilution test systems facilitate reading of MICs and are often considered as gold standards for susceptibility testing. However, susceptibility testing by the disc diffusion method has the advantages of simplicity, low cost and a high degree of flexibility in the selection of agents tested (Richter & Ferraro, 2007). Our results show that rifaximin – despite its water-insolubility – may be a suitable candidate for disc diffusion testing. Whether 40 µg per disc is the ideal concentration must be examined in further studies. Whether it was prudent to officially license a substance ‘for treatment of all enteric infections caused by susceptible organisms’ also remains to be answered.
